# Predicting the response of localised oesophageal cancer to neo-adjuvant chemoradiation

**DOI:** 10.1186/1477-7819-5-97

**Published:** 2007-08-23

**Authors:** Charles M Gillham, John Reynolds, Donal Hollywood

**Affiliations:** 1 Academic Unit of Clinical and Molecular Oncology, St James's Hospital, Dublin, Ireland

## Abstract

**Background:**

A complete pathological response to neo-adjuvant chemo-radiation for oesophageal cancer is associated with favourable survival. However, such a benefit is seen in the minority. If one could identify, at diagnosis, those patients who were unlikely to respond unnecessary toxicity could be avoided and alternative treatment can be considered. The aim of this review was to highlight predictive markers currently assessed and evaluate their clinical utility.

**Methods:**

A systematic search of Pubmed and Google Scholar was performed using the following keywords; "neo-adjuvant", "oesophageal", "trimodality", "chemotherapy", "radiotherapy", "chemoradiation" and "predict". The original manuscripts were sourced for further articles of relevance.

**Results:**

Conventional indices including tumour stage and grade seem unable to predict histological response. Immuno-histochemical markers have been extensively studied, but none has made its way into routine clinical practice. Global gene expression from fresh pre-treatment tissue using cDNA microarray has only recently been assessed, but shows considerable promise. Molecular imaging using FDG-PET seems to be able to predict response after neo-adjuvant chemoradiation has finished, but there is a paucity of data when such imaging is performed earlier.

**Conclusion:**

Currently there are no clinically useful predictors of response based on standard pathological assessment and immunohistochemistry. Genomics, proteomics and molecular imaging may hold promise.

## Background

Cancer medicine is in the midst of a technological revolution and the way the disease is managed is undergoing enormous change. For the very first time it is becoming increasingly possible to individualise a patient's treatment by predicting those that will and those that will not respond to a chosen therapy. This is being achieved through rapid developments in both advanced diagnostic imaging and translational medicine. Clinical trials incorporating expression array data are already underway in some of the more common tumour sites, such as breast cancer [1]. As a result the foundations have been laid for some of the less frequent, but by no means less serious, pathological types.

Oesophageal cancer is the eighth most common cancer worldwide and more than 80% of cancers occur in less developed countries [2]. The incidence in Europe is 5.4 per 100,000 per year with approximately 4.9 deaths per 100,000 per year. Survival correlates with stage of disease. Five-year survival rates range from 40 to 62 percent for patients treated for localized cancer (stage I and IIA), and from 18 to 25 percent for those with involvement of regional nodes (stage IIB and III) [3]. According to data from the National Cancer Institute Surveillance, Epidemiology and End Results (SEER) Program, the five-year survival rate for all patients with oesophageal cancer has improved modestly over the last 30 years, from 5 percent in the years 1974 to 1976, to 13 percent during the period 1992 to 1998 [4]. These dismal figures are indicative of the advanced stage of disease (local-regional or metastatic, stages IIB, III and IV) at diagnosis in most individuals [4].

Although the incidence of squamous cell carcinoma has been declining over the past two decades, the incidence of adenocarcinoma has increased markedly. In the US adenocarcinoma now accounts for more than 50% of oesophageal cancer cases [5]. The high mortality rate reflects early lymphatic and haematogenous spread as well as the lack of effective treatments and early therapeutic options.

For many years the standard therapy for localised oesophageal carcinoma has been surgical resection [6]. However local control and overall survival remain poor, and even after radical resection and lymphadenectomy the 5-year survival is at best approximately 40 percent [7,8]. In an effort to improve these figures the management of local-regional oesophageal cancer has undergone a major evolution over the past 15 years. Numerous strategies employing various pre- and post-operative therapies have been studied as well as trials where surgery has been omitted altogether.

To date the optimal therapy for potentially resectable oesophageal cancer remains unclear.

Although somewhat controversial, the use of neo-adjuvant chemoradiation (CRT) has increased outside of clinical trials, and the Patterns of Care studies in the US showed that preoperative CRT therapy increased from 10.4% during 1992–1994 to 26.6% in 1996–1999 for patients with stage IIb and III oesophageal cancer [9]. The same is now true in many European Centres.

**Figure 1:**
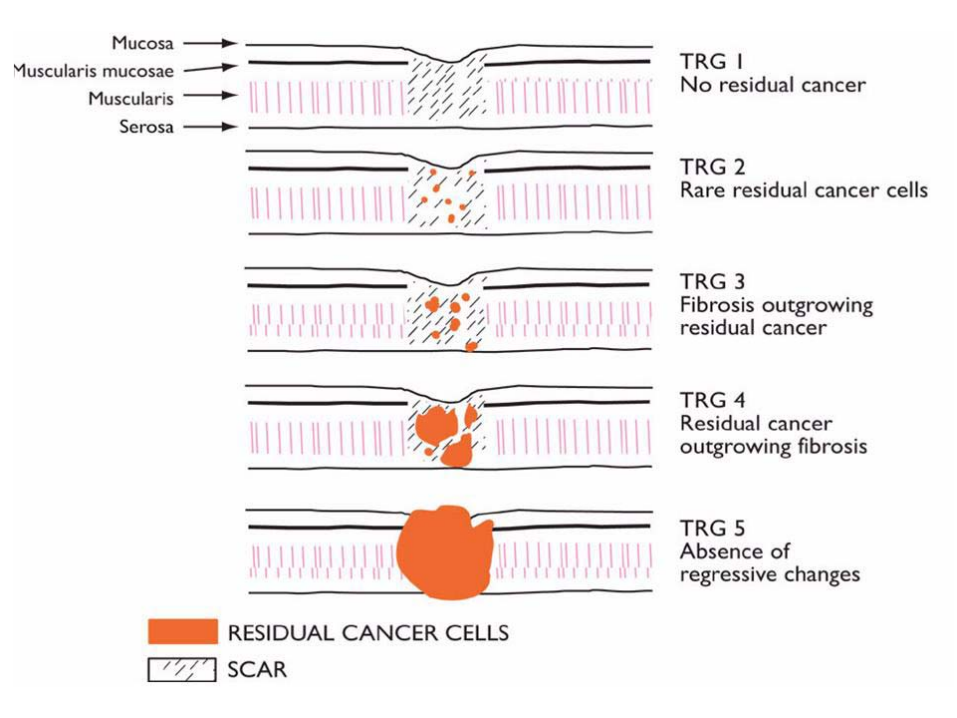
Pathological response grading following neoadjuvant chemoradiation in oesophageal cancer (Mandard [11]).

However, many patients do not benefit from such an approach and there are now evolving strategies to identify predictive response markers. Several analyses suggest that it is the *response *to preoperative therapy (particularly the absence of residual disease (pCR) in the surgical specimen) that best predicts disease-free and overall survival [10]. A pCR occurs in approximately 15–30% of cases, and three-year survival rates of approximately 60% irrespective of the applied protocol, type of histology and tumour stage are achieved [10]. A further subdivision of pathological response to neoadjuvant regimens, the tumour regression grade (TRG), may also identify patterns of incomplete response that may impact on treatment outcome [11]. Regression grading stratifies response based on the biological effect of radiation on tumours, dividing it into 5 different grades based on the ratio of fibrosis to tumour (Figure 1). Mandard described complete response as histologic fibrosis with or without inflammation extending through the different layers of the oesophageal wall, but with no viable residual tumour cells (tumour regression grade (TRG) 1). Subtotal response (TRG 2) was characterized by the presence of rare residual cancer cells scattered through the fibrosis. An increase in the number of residual cancer cells, but with fibrosis predominating was termed a partial response (TRG 3). Minimal remission (TRG 4) showed residual cancer outgrowing fibrosis. Absence of any regressive changes (TRG 5) defined no change.

The addition of the pathological response to standard pTNM has been recently advocated [12]. Where a cohort of patients may benefit from neoadjuvant CRT, with pCR and TRG the surrogate markers, many patients will not be helped, and their prognosis may be worsened by delay in surgery and by the added risks of surgery in patients on multimodal protocols. A predictor of response or resistance based on pre-treatment demographics, imaging, histolopathologic, molecular, or genetic information would have potentially enormous application in optimising outcomes and in the design of clinical trials. However, despite numerous studies to date no clear candidate markers that predict pathological response have emerged.

## Predicting response

### Conventional patient and histological indices

Numerous clinical and pathological parameters have been analysed in a small number of oesophageal cancer studies with regard to their utility in the prediction of the response to pre-operative CRT. Mandard found that the larger the initial primary tumour the poorer the overall response to neo-adjuvant treatment [11]. Pre-treatment performance status, primary location and age are clearly important factors in terms of tolerating therapy, but they are not known to be associated with the pathological response [13].

Other pre-therapy parameters that some have identified as potentially useful include the patient's nutritional status [14], tumour cell aneuploidy [15] and tumour differentiation [16].

In general, however, many of these factors tend to be relatively crude determinants of the overall management approach, i.e. curative or palliative, rather than being predictors of the molecular *response *to treatment. It would seem that it is the post-therapy pathological stage that best predicts the survival of patients who receive neo-adjuvant CRT [13] and more precise markers are required in order to determine the most appropriate therapeutic strategy.

### Tissue markers

Most studies have correlated the expression of molecular markers in the pre-treatment biopsy with either the biological response to treatment in the oesophagectomy specimen or to survival/recurrence data following treatment.

These markers have usually been identified by immuno-histochemical means.

### Apoptosis

#### P53

**Figure 2:**
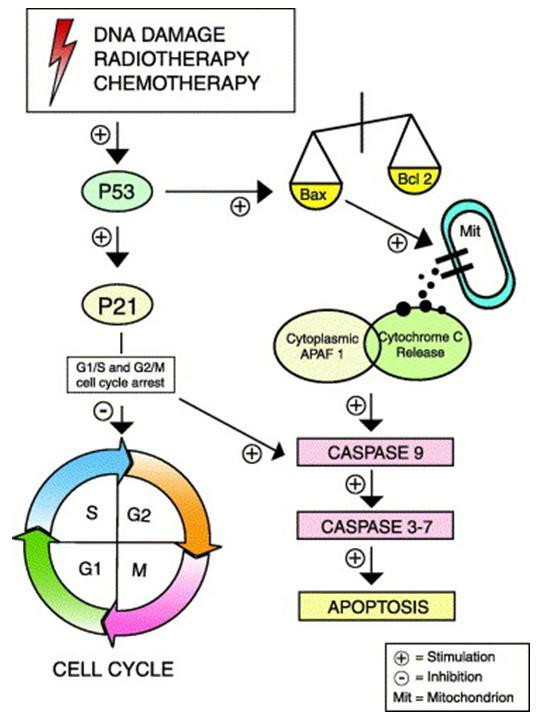
Flow diagram of the P53/apoptosis pathway. Constituents of this pathway are the most commonly assessed predictive markers in oesophageal cancer [19].

The p53 gene is one of the most widely investigated in human cancer, including oesophageal. Several groups have found that the protein it encodes is one of the prognostic indicators in various cancers [17,18]. It is one of the genes responsible for repairing a damaged cells' DNA or triggering apoptosis when this cannot occur (Figure 2[19]) and it is generally accepted that it may be intrinsically involved in the response to CRT [20,21]. Several trials have studied p53 expression as a determinant of response to chemotherapy with or without radiotherapy in oesophageal cancer. In oesophageal adenocarcinoma Duhaylongsod immunostained 42 patients for p53 and c-erb B2 protein. All patients received neoadjuvant CRT followed by resection [22]. They found that 84% of the p53 positive tumours had residual disease as opposed to 44% of the p53 negative (p = 0.01). Similarly in patients with squamous cell carcinoma Seitz *et al*., identified immunohistochemically a significant association between p53 overexpression and a lower complete response [15]. To counter these results other groups have found no such an association [23]. It may be the small sample sizes or the differences in immunohistochemical staining methods that explain these discrepancies. Equally it has been postulated that p53 overexpression is not necessarily synonymous with p53 mutations [24]. Furthermore absence of p53 staining may occur with gene deletion, failure of transcription, or a non-stabilizing mutation, all of which may be associated with loss of p53 function [25].

#### P21

The p21 protein is a key member of the p53 signalling pathway (figure 2). It is transcriptionally activated by p53 following DNA damage by ionising radiation, which in turn causes cell cycle arrest, and apoptosis [26,27]. It has been studied as a response predictor because it disrupts regulatory networks, in particular those involved in cell death signaling. It may therefore be a causative factor of radioresistance.

Nakamura *et al*., found that the survival of patients with p21 positive oesophageal tumours treated with definitive CRT was significantly better than those where no such expression existed (p = 0.0013) [28]. They also identified that the survival of those patients with p53 negative tumours was significantly higher if they were p21 positive than negative (P = 0.0452).

Conversely another Japanese group found that whilst p21 positive expression in the absence of p53 was associated with favourable effects from preoperative *chemotherapy *there was no such correlation between p21 expression and the clinical effects following *CRT *[29,30].

#### Survivin

Survivin is a member of the inhibitor of apoptosis family and is known to be involved in resistance to chemo- and radiation therapy. It is expressed in several cancers particularly rectal cancer where it's expression is associated with a poor survival following CRT [31]. In oesophageal cancer Kato *et al*., found that that high survivin expression predicted a significantly reduced median survival (9.0 vs 30.0 months, p = 0.0023) in patients receiving pre-operative chemotherapy [32]. Conversely other groups have found the reverse. In tissue samples taken from patients prior to CRT elevated tumour:normal levels of survivin mRNA were significantly associated with improved survival but not histological regression [33].

#### Cyclo-oxygenase-2 (COX-2)

COX-2 plays an important role in prostaglandin synthesis and mediates angiogenesis and tumour growth. It is overexpressed in various human malignancies and is an important mediator of tumour invasiveness and metastasis [34]. In addition, clinical studies in nasopharyngeal [35] and cervical.

Cancer [36] have demonstrated that endogenous COX 2 expression in pre-treatment biopsies are indicative of a poor response to and an unfavourable prognosis following chemotherapy and ionising radiation and chemotherapy. There is similar data in oesophageal cancer. In 29 biopsies taken from squamous cell carcinoma patients who went on to receive CRT high Cox-2 mRNA levels were significantly associated with a poor response to treatment (p < 0.05) [37]. Whilst Cox-2 levels have not been correlated with response to neoadjuvant CRT in oesophageal adenocarcinoma there is a wealth of literature showing that high levels of expression in this subtype are associated with aggressive disease and a poor survival [38].

### Tumour hypoxia

Hypoxic regions within tumours may lead to chemo- and radioresistance by depriving cells of oxygen necessary for the cytotoxic activities of these agents [39]. Furthermore, tumour hypoxia promotes up-regulation of angiogenic and tumour cell survival factors resulting in increased proliferation, radioresistance and angiogenesis. Angiogenesis has an important role in solid tumour growth and metastasis [40]. Vascular endothelial cell growth factor (VEGF) is the main angiogenic factor known to be involved in pathological angiogenesis. Its induction in several solid tumours is thought to be important with respect to the chemotherapy and radiotherapy response [41]. In one study CRT was administered to 52 patients with oesophageal squamous cell carcinoma [42]. Expression of p53, thymidine phosphorylase and VEGF was analysed by immuno-histochemistry. Sixty percent then underwent radical surgery and from these multivariate analysis identified that only VEGF was a significant prognostic indicator (p = 0.0147). Its expression was associated with a high incidence of treatment failure and a significantly worse 5-year survival rate (p = 0.037). These results are further supported by Gorski *et al*., who found that the anti-tumour effects of ionizing radiation could be enhanced if VEGF activity was blocked [43]. It remains unclear, however, as to whether the expression of VEGF by itself directly or indirectly determines whether a tumour responds to CRT [44].

### Growth regulation

#### Epidermal growth factor receptor (EGFR)

Aberrant activation of the epidermal growth factor receptor (EGFR) is frequently observed in neoplasia, notably in tumours of epithelial origin. In squamous cell oesophageal carcinoma Hickey *et al*., compared tumour response with expression of EGFR and proliferating cell nuclear antigen [45]. There was a significant survival advantage in those staining negative for one or both markers, while those which stained positive responded poorly. The same would appear to be true for oesophageal adenocarcinoma. Not only was EGFR expression associated with a higher TNM stage, but also with shorter disease-free and overall survival [46].

#### HER-2

HER-2 protein is a 185 kD transmembrane protein and a member of the EGFR family. It is a proto-oncogene that encodes a tyrosine kinase growth factor receptor and has been associated with the pathogenesis of several human cancers. Its over-expression in breast cancer is associated with a poorer prognosis [47]. In oesophageal cancer the data is conflicting. In adenocarcinoma Duhaylongsod *et al*., found that over-expression predicted a favourable response to CRT and a 5 year actuarial survival of 60% [22]. However, other groups have found that over-expression in this sub-type was associated with a poor prognosis [48]. In squamous cell carcinoma Akamatsu *et al*., however, found that immunostaining was useful for predicting chemoradioresistance but this did not correlate with survival [49].

#### Cyclins

The cyclins are important oncogenic proteins and regulators of the cell cycle.

Sarbia *et al*., assessed cyclin D1 expression by immuno-histochemistry in squamous cell oesophageal cancer [50]. They identified that in patients treated with multi-modal therapy cyclin D1 expression correlated with a poor response to treatment but not to overall survival. Cyclin E is reportedly overexpressed in adenocarcinoma of the distal oesophagus and in gastric cancer is associated with reduced survival [51]. In oesophageal cancer, however, its prognostic effect has not been elucidated.

### Markers of resistance to commonly used chemotherapy agents

Platinum-based compounds, 5-fluorouracil and taxanes are the agents most commonly used in the treatment of oesophageal cancer and advances in molecular pharmacology have enhanced our understanding of their mechanisms of action and modes of resistance.

Joshi *et al*., measured gene expressions of thymidylate synthase 1 (TS1), glutathione S-transferase π (GSTP1), and excision cross-complementing gene 1 (ERCC1) by quantitative RT-PCR in the pre-treatment biopsies of tumour tissue specimens taken from patients scheduled to receive neoadjuvant 5-FU, cisplatin and radiotherapy. Elevated expression of these genes was significantly associated with a poor survival (p = 0.007) [52].

#### Metallothionein (MT)

MT is a small protein with a high affinity for divalent heavy metal ions. It is involved in many patho-physiological processes, like metal homeostasis and detoxification, cell proliferation, apoptosis, therapy resistance, and protection against oxidative damage. Alterations in the immuno-histochemical expression of MT have been reported for various human tumours, and a high expression has been found to be associated with a poor clinical outcome [53]. Much of the work in squamous cell oesophageal cancer comes from Japan. MT-positivity in patients treated with neoadjuvant chemotherapy with or without radiotherapy has usually been associated with a worse prognosis [54]. Some studies have, however, shown no such association [55]. There is no data on the effects of MT-positivity and the response to treatment in oesophageal adenocarcinoma, but it is implicated in the malignant transformation of Barrett's epithelium [56].

#### Nuclear factor-kappa B (NF-KB)

NF-KB regulates several genes involved in inflammatory, immune and apoptotic responses. In patients treated with neoadjuvant CRT for oesophageal adenocarcinoma Abdel-Latif *et al*., identified that its' expression was inversely related to a major or complete pathological response. 75% of those that did not respond were NFKB negative, whilst only 18% of the responders were positive (p < 0.00001) [57]. More recently Izzo *et al*., found that activated NF-KB expression was significantly associated with residual disease following neo-adjuvant CRT (p = 0.006), metastatic progression (p = 0.01) and reduced survival (p = 0.01) in 80 oesophageal cancer patients [58].

## Serum markers

Serum markers have not proved particularly useful in predicting the response of oesophageal cancer to neo-adjuvant therapy.

Kim *et al*., evaluated serial CEA levels in 90 patients with potentially resectable oesophageal and gastric adenocarcinoma treated with preoperative chemotherapy [59]. Measurements were taken before treatment and serially thereafter. An increasing CEA level predicted relapse and correlated well with visceral involvement and clinical responses correlated with declining levels of CEA. However, the levels did not predict resectability or survival.

Another group analysed serum VEGF levels in patients with oesophageal cancer before, during and after CRT. Levels did not decline during therapy. They fell following resection but then rose to pre-operative values before falling to normal at three months. They postulated that the tumours were not generating VEGF and therefore levels could not be used as response markers [60].

Quillien *et al*., examined the serum markers CYFRA 21-1, TPA and SCC in 96 patients with squamous cell oesophageal carcinoma. CYFRA 21-1 was the only marker whose pre-treatment levels significantly correlated with pathological response, but on multivariate analysis treatment was the only independent factor [61].

Nakamura *et al*., assessed the clinical value of CYFRA 21-1 in comparison to SCC-Ag, CEA and CA19-9 in 112 patients with squamous cell carcinoma. Levels of CYFRA 21-1 correlated closely with stage and with clinical response to both chemotherapy and CRT [62].

These reports suggest that CYFRA 21-1 may be the most useful serum marker currently available, but this has not become widely adopted.

### Gene expression arrays

Patients diagnosed with the same stage of cancer by conventional clinical and histopathological criteria may have a completely different course of disease. Since cancer is fundamentally a malfunction of gene expression giving rise to aberrant malignant growth, the most direct classification approach would be to analyse gene expression patterns. To find the relatively small number of genes that are characteristically de-regulated in a given cancer cell, among thousands of genes that are normally expressed, requires high-throughput technologies and sophisticated computational tools.

The first high density microarrays were developed to analyse gene expression by quantitating thousands of mRNAs present in a cell or tissue sample (DNA arrays). Other microarray approaches include the quantitation of proteins (protein arrays), or the analysis of a large number of tissue samples in parallel (tissue arrays). It was clear early on that arrays could be very useful tools in molecular profiling of cancer cells, thus revealing information that cannot be obtained by traditional histological assessment [63].

Over the last few years there have been numerous gene expression studies that have enhanced our understanding of the biology of oesophageal cancer [64-70]. It was hoped that these might identify potential biomarkers for therapeutic targeting, but none of them specifically addressed treatment and pathological outcome data and so their clinical value is so far limited.

There has, however, been only one clinically relevant study. The MD Anderson performed gene expression analyses on 19 patients prior to neo-adjuvant CRT and correlated their findings with the final histopathological response [71]. Unsupervised hierarchical cluster analysis of the cancer biopsies segregated them in to two molecular subtypes. Amongst the adenocarcinomas, most that achieved a complete response clustered in one group and all but one of the poorer responders in the other. They identified a number of genes that were differentially expressed between the two molecular sub-types, several of which have been reported to occur in oesophageal cancer. The authors stress that this was a preliminary study and clearly larger sample numbers and stringent validation is essential before the data generated from array experiments can be evaluated in clinical trials.

## Imaging

Despite their widespread use in primary staging, conventional computed tomography (CT) and endoscopic ultrasound (EUS) have not proven beneficial in predicting the response of oesophageal tumours to CRT [72,73]. With the advent of molecular imaging, which has demonstrated superiority over conventional imaging techniques at diagnosis, FDG-PET (^18^fluorodeoxyglucose positron emission tomography) is frequently being used to predict response in several malignancies [74,75].

Alterations in tissue metabolism often precede anatomical changes and this forms the basis of FDG-PET scanning. In 22 patients with advanced breast cancer, changes in FDG uptake were able to predict the eventual histopathologic response with an accuracy of 88% after the first course of drugs and 91% after the second course [76]. Similar utility has been described in cancers of the lung [77] and colon [78], as well as Hodgkin's [79] and non-Hodgkin's lymphoma [80].

There has been considerable work in oesophageal cancer. Studies evaluating tumour response with PET during and at the completion of neo-adjuvant therapy have yielded encouraging results (Table 1). These studies suggest that changes in FDG uptake in response to therapy correlate with the pathological response as well as predict the risk of local recurrence and survival. Squamous cell oesophageal cancer is more frequently treated with neoadjuvant *CRT*. Studies have therefore been performed in either squamous cell carcinoma exclusively or in a combination of the two tumour types. There have been no studies investigating the predictive role of FDG-PET in oesophageal adenocarcinoma exclusively and consequently it is not possible to ascertain the usefulness of molecular imaging in predicting response to CRT in one subtype over another.

**Table 1:** Studies that have assessed the role of FDG-PET in predicting the response of oesophageal cancer to neoadjuvant chemoradiation

Study + Reference	Path	Chemo	Radiotherapy	Second PET	Main Results
Brucher et al, 2001 [82]	24 SCC	5 FU	30 Gy/15#	3 weeks after CRT	An SUV reduction of >52% led to sensitivity, specificity, positive and negative predictive values of 100%, 55%, 72% and 100% respectively
Kato et al, 2002 [83]	10 SCC	cis/5 FU	40 Gy/20#	2 weeks after CRT	Pathological response did not correlate with rate of reduction of SUV
Arslan et al, 2002 [84]	22 AD 2 SCC	See notes^‡^	40–50.4 Gy/20–28#	4 wks after CRT	Change in volume identified responders. Quantitative evaluation of primary tumour pre and post therapy could not separate post therapy inflammation from residual tumour
Flamen et al, 2002 [85]	27 SCC 9 AD	cis/5 FU	40 Gy/20#	4–6 weeks after CRT	When >80% reduction in FDG tumour:liver uptake ratios used to define response sensitivity 71% and specificity 82%
Downey et al, 2003 [86]	26 AD 13 SCC	cis/taxol	50.4 Gy/28# (2 had no RT)	After CRT (not specified)	SUV reduction >60% associated with non-significant disease-free and survival advantage compared to when reduction <60%
Brink et al, 2004 [87]	13 AD 7 SCC	cis/5 FU	36 Gy/20#	2–3 weeks after CRT	No correlation
Swisher et al, 2004 [88]	73 AD 10 SCC	See notes*	50.4 Gy/28#	After CRT (not specified)	Pathological response correlated with post therapy SUV. Post therapy SUV > 4 was only pre-operative factor to correlate with decreased survival
Wieder et al, 2004 [90]	38 SCC	5 FU	40 Gy/20#	During CRT in 27	Changes in SUV were significantly different between 2 groups
Song et al, 2005 [89]	32 SCC	cis/cape	45.6/38#(BID) + 46 Gy/23#/5 wks	4 weeks after CRT	Pathological response could be predicted when analysis limited to initial highly metabolic tumours
Levine et al, 2006 [81]	52 AD 9 SCC 3 UN	cis/5 FU	50.4 Gy/28#	After CRT (not specified)	Reduction in SUV ≥ 10 associated with significant response
Gillham et al, 2006 [91]	29 AD 3 SCC	cis/5 FU	40.05 Gy/15# 44 Gy/22#	After 1 week of CRT	Changes in SUV during treatment did not predict pathological outcome

Key: Path – histological sub-type, AD – adenocarcinoma, SCC – squamous cell carcinoma, UN – undifferentiated, CT – computed tomography, 5 FU – 5-fluorouracil, Gy – Gray, # – fractions of radiotherapy, cis – cisplatin, LV – leucovorin, carbo – carboplatin, cape – capecitabine, gem – gemcitabine, BID – twice daily fractionation

^‡ ^Received cisplatin/5 fluorouracil or cisplatin/taxol or carboplatin/5 fluorouracil in combination with radiotherapy

*Patients received either irinotecan/5 FU/docetaxol (up to 2 cycles) prior to CRT with same drugs (reduced dose) or same RT with cisplatin/5 FU or taxol/carboplatin (no pre-CRT chemotherapy)

Levine *et al*., performed an FDG-PET at diagnosis and following CRT in 31 oesophageal cancer patients [81]. They found that the standardized uptake value (SUV) decreased significantly more in those patients who responded (pathological complete response or microscopic residual disease) than in those who did not (p = 0.05). The MD Anderson study performed CT, EUS and PET before and after CRT in 73 adenocarcinoma and 10 squamous cell carcinoma patients [73]. They found that PET most accurately predicted long-term survival and that by uni- and multivariate Cox regression analysis an SUV ≥4 had the greatest accuracy in predicting pathological response. Similarly there is some evidence to suggest that the *initial *SUV may be predictive of outcome. Levine's group found that an SUV at diagnosis ≥15 was associated with an observed 77.8% significant response (pathological complete response or microscopic residual disease) compared with 24.2% for patients with a pretreatment SUV < 15 (P = 0.005).

In the majority of studies to date the second PET has been performed *after *the neo-adjuvant phase of treatment [81-89]. Earlier response prediction, by repeating the PET *during *neoadjuvant therapy, could potentially differentiate responders from non-responders, minimise the inherent toxicity associated with current regimens and direct non-responders towards alternative therapies. Only two such studies have been performed [90,91]. Wieder *et al*., reported pre-therapy and early repeat FDG-PET scans at 2 weeks (of a four week neo-adjuvant CRT regime) in 27 patients with squamous cell carcinoma of the oesophagus, a treatment regimen similar to that which was used in the Gillham *et al*., study. In the former, the change in SUV following CRT reliably separated responders and non-responders; using a reduction in SUV of 30% as the optimal cut off point, they identified a sensitivity and specificity of 93% and 88% respectively with a satisfactory accuracy of 79%. In the latter study the second PET scan was performed after only one week of CRT and included both squamous cell and adenocarcinoma sub-types. Changes in FDG uptake failed to predict the pathological response. Both studies were small and are not directly comparable. However the inflammatory effect of ionising radiation on oesophageal tissue may interfere with the interpretation of the second PET scan.

## Future

For many years formalin fixed paraffin embedded tissue samples have provided a wealth of information using immuno-histochemical and DNA based techniques. The process is relatively straightforward and inexpensive and the material can be stored for a number of years. A number of different markers have been identified that seem to be associated with a good or a poor response to treatment in oesophageal cancer. However, comparing the results of different studies is markedly hampered by the differing individual techniques and batch-to-batch variability [92]. As a result none of them have found their way into routine clinical use. Some of these issues may be overcome by the use of tissue microarrays, but such an approach remains very much in its infancy until the appropriate candidate markers are identified.

Advances in microarray technology may mean that, by assessing the transcriptional activity of a large number of genes, the complex gene expression profile may contain more information than any individual molecule that contributes to it. A number of studies have used such an approach to study the different profiles between oesophageal cancer and its pre-malignant components. Unlike in other tumour sites only one study has specifically addressed the issue of response prediction to CRT in oesophageal cancer [71].

The other area of translational medicine that holds promise is that of serum proteomics. It is based on the assumption that cancers shed protein debris into the bloodstream. The technique has been used to differentiate benign from malignant disease [93-95] but, to date, has not been applied in the area of response prediction.

## Conclusion

The management of patients with localised oesophageal cancer would be greatly enhanced if predictors of response could be identified.

It would seem there is little to be gained by studying conventional patient and histological indices. At present none of the tissue or serum markers of response to neo-adjuvant treatment are sufficiently accurate on their own to be used to predict response in an individual patient. Genomics and proteomics are fast generating vast amounts of data. In time, it seems likely that these may lead to the detection of stringently validated markers, which become part of routine diagnostic work-up.

Molecular imaging is another evolving science. The cumulative data suggests that changes seen on serial PET scans after neo-adjuvant therapy correlate with the final pathological response and survival. However larger numbers are required and it would be more clinically beneficial if such imaging proved predictive earlier in the course of treatment.

## Competing interests

The author(s) declare that they have no competing interests.

## Authors' contributions

CG conceived the idea and wrote the manuscript.

JR and DH were involved in the drafting and final approval of the manuscript

All authors read and approved the final manuscript.
